# Integrating the characteristic genes of macrophage pseudotime analysis in single-cell RNA-seq to construct a prediction model of atherosclerosis

**DOI:** 10.18632/aging.204856

**Published:** 2023-07-08

**Authors:** Zemin Tian, Shize Yang

**Affiliations:** 1 Department of Vascular and Thyroid Surgery, The First Affiliated Hospital of China Medical University, Shenyang 110001, Liaoning, China; 2 Department of Thoracic Surgery, The First Affiliated Hospital of China Medical University, Shenyang 110001, Liaoning, China

**Keywords:** atherosclerosis, macrophage, pseudotime analysis, phenotypic transformation

## Abstract

Background: Macrophages play an important role in the occurrence and development of atherosclerosis. However, few existing studies have deliberately analyzed the changes in characteristic genes in the process of macrophage phenotype transformation.

Method: Carotid atherosclerotic plaque single-cell RNA (scRNA) sequencing data were analyzed to define the cells involved and determine their transcriptomic characteristics. KEGG enrichment analysis, CIBERSORT, ESTIMATE, support vector machine (SVM), random forest (RF), and weighted correlation network analysis (WGCNA) were applied to bulk sequencing data. All data were downloaded from Gene Expression Omnibus (GEO).

Result: Nine cell clusters were identified. M1 macrophages, M2 macrophages, and M2/M1 macrophages were identified as three clusters within the macrophages. According to pseudotime analysis, M2/M1 macrophages and M2 macrophages can be transformed into M1 macrophages. The ROC curve values of the six genes in the test group were statistically significant (AUC (IL1RN): 0.899, 95% CI: 0.764-0.990; AUC (NRP1): 0.817, 95% CI: 0.620-0.971; AUC (TAGLN): 0.846, 95% CI: 0.678-0.971; AUC (SPARCL1): 0.825, 95% CI: 0.620-0.988; AUC (EMP2): 0.808, 95% CI: 0.630-0.947; AUC (ACTA2): 0.784, 95% CI: 0.591-0.938). The atherosclerosis prediction model showed significant statistical significance in both the train group (AUC: 0.909, 95% CI: 0.842-0.967) and the test group (AUC: 0.812, 95% CI: 0.630-0.966).

Conclusions: IL1RN^High^ M1, NRP1^High^ M2, ACTA2^High^ M2/M1, EMP2^High^ M1/M1, SPACL1^High^ M2/M1 and TAGLN^High^ M2/M1 macrophages play key roles in the occurrence and development of arterial atherosclerosis. These marker genes of macrophage phenotypic transformation can also be used to establish a model to predict the occurrence of atherosclerosis.

## INTRODUCTION

Atherosclerosis (AS) is a local chronic inflammatory disease of the artery wall [1]. In AS, the inflammatory response is maintained for decades by the inflow, proliferation, and activation of immune cells [2, 3]. Among the many types of immune cells, macrophages have attracted great interest in AS because of their complex functions and numerous subtypes [4, 5]. Under the stimulation of the atherosclerotic microenvironment, macrophages can polarize into different types of macrophages, which involves changes in gene expression profile and cell functions [6]. In previous studies, polarized macrophages were mainly divided into M1 macrophages and M2 macrophages according to their phenotype and functions [7, 8]. M1 macrophages, as proinflammatory macrophages, can release a variety of chemokines and proinflammatory cytokines, including CCL2 (MCP-1), CCL3 and IL-1β, IL-6, IL-12, IL-23 and TNF, which can induce inflammatory reactions and can also secrete reactive oxygen intermediates (ROI), nitric oxide (NO) and lysosomal enzymes to kill and remove pathogens. However, ROS can induce tissue damage, leading to irreparable tissue damage, which may promote the formation of atherosclerosis and reduce the stability of atherosclerotic plaques [9]. In contrast, activated macrophages (M2) play a preventive role in the progression of human and mouse atherogenesis. M2 macrophages, as anti-inflammatory macrophages, mainly secrete anti-inflammatory cytokines, typically including IL-10 and TGF-β [10, 11]. These factors counteract the proinflammatory effects caused by M1 macrophages, thus promoting tissue repair and reducing tissue damage. In atherosclerotic plaques, M2 macrophages can inhibit the formation of atherosclerosis and maintain plaque stability [12, 13].

With the improved understanding of atherosclerosis by researchers, traditional lipid-lowering and anti-inflammatory treatments are being replaced by targeted treatments [14]. Research on the prevention and treatment of atherosclerosis has also become refined in this era [14]. These technologies include nanotechnology drug treatment, regulation of the ratio of M1/M2 macrophages, control of macrophage phenotype conversion, and regulation of the plaque microenvironment [8].

In this study, we analyzed the phenotypic transformation of macrophages in atherosclerosis through pseudotime analysis. We identified a class of macrophages that simultaneously express M1 and M2 macrophage marker genes and can transform into M1 or M2 macrophages. Finally, we selected the genes with the most obvious differential expression to build an atherosclerosis prediction model based on the results of macrophage pseudotime analysis. These genes may provide new ideas for the targeted treatment of atherosclerosis.

## MATERIALS AND METHODS

### Bulk sequencing data processing

We downloaded GSE43292 (training group) and GSE28829 (test group) from the GEO database (https://www.ncbi.nlm.nih.gov/). We used the “WGCNA” package [15] to screen disease-characteristic genes from GSE43292. We utilized the “limma” package and the “VennDiagram” package to identify 223 intersecting genes (Supplementary Table 5) obtained from macrophage cell trajectory analysis. We then took the intersection of these genes with the 1086 AS-characteristic genes (Supplementary Table 4) obtained from GSE43292, resulting in 50 macrophage-related genes (Supplementary Table 6). Next, we compared the residual value and AUC curve of random forest (RF) and support vector machine (SVM) models. Finally, we used the RF method to select disease-characteristic genes again. Independent heatmaps of intersecting genes were drawn in the training and test groups. We selected the important genes with an RF score>1 in the training group and drew the AUC (area under the curve) curve in the test group for verification. Next, we used these genes to construct a disease prediction model. The “rms” and “rmda” packages [16] were used to draw the nomogram, calibration curve, decision curve analysis (DCA) [17], and clinical impact curve of DEGs predicting atherosclerosis in patients.

### scRNA sequencing data processing

We downloaded the GSE159677 single-cell dataset from the GEO database (https://www.ncbi.nlm.nih.gov/). We removed cells with fewer than 200 genes, more than 7,000 genes, or more than 10% mitochondrial genes. Analysis was performed on 49576 filtered cells. Using the “LogNormalize” method, gene expression was normalized and scaled. In each sample, the top 2000 highly variable genes (HVGs) were identified using the “vst” method after data normalization. After identifying significant principal components (PCs), PCA was applied. Batch correction was performed using the “Harmony” R package (version 0.1.0) to avoid batch effects resulting from sample identity that could disrupt downstream analysis. Finally, 50 PCs were selected for t-distributed stochastic neighbor embedding (t-SNE) analysis. We set “FindClusters” with a resolution of 2.0 to divide cells into 45 different clusters, which were divided into 9 cell types, and conducted manual inspection according to the results of “FindAllMarkers”.

We used the same method to set “FindClusters” to divide macrophages into 13 different clusters with a resolution of 0.05, divided these clusters into three cell types with marker genes, and used the results of “FindAllMarkers” for manual inspection. The results of uniform manifold approximation and projection for dimension reduction (UMAP) were visualized, and a heatmap of macrophage subtypes was drawn.

We analyzed the trajectory of M1 macrophages and M2 macrophages with the “Monocle” package [18] and drew a heatmap. We used the same method to analyze M2 macrophages and M2/M1 macrophages, M1 macrophages and M2/M1 macrophages.

### Consent for publication

The authors agree for publication.

### Availability of data and materials

The data for this study were derived from the GEO database (https://www.ncbi.nlm.nih.gov/geo/).

## RESULTS

### Analysis the heterogeneity of macrophages in the atherosclerotic single-cell transcriptome

**Figure 1:**
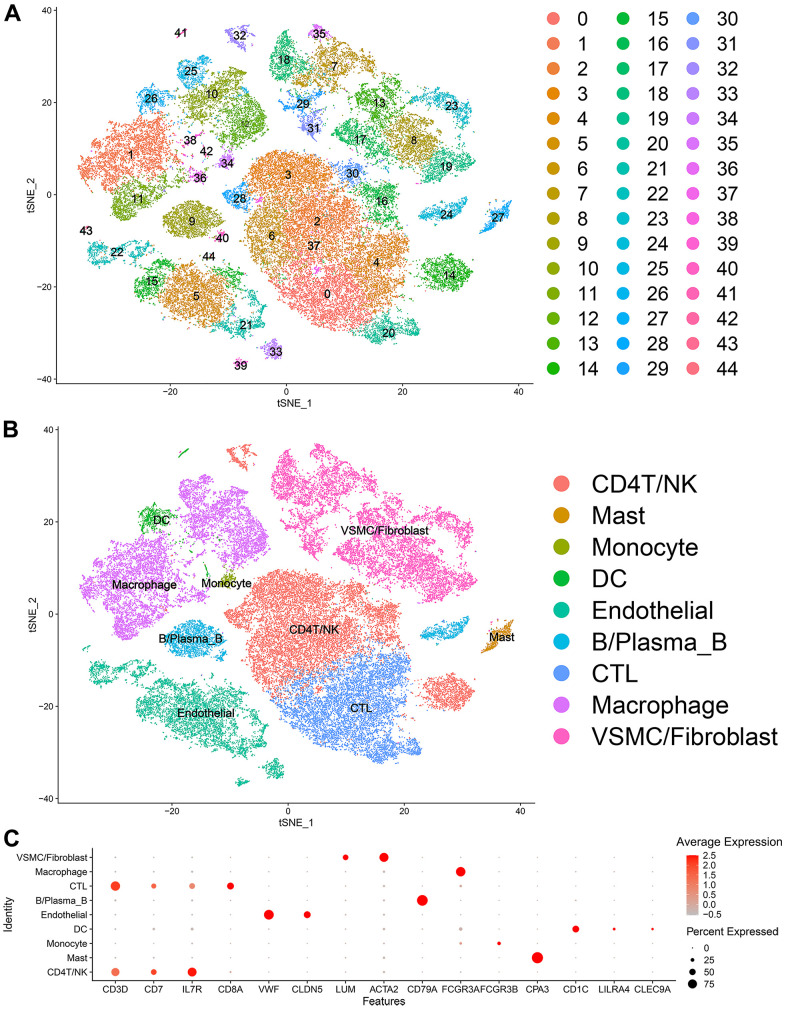
Single-cell transcriptome data: (**A**, **B**) The single cells were divided into 45 groups on the basis of their transcriptome data and then ultimately divided into 9 cell populations. (**C**) Circle chart: The X-axis represents the marker genes that define the cells, and the Y-axis represents the different cell populations.

Forty-five clusters could be assigned to known cell lineages according to the marker genes defined in a previous study (Figure 1A and Supplementary Table 1). We used t-SNE analysis to visualize the 9 clusters (Figure 1B) and identify marker genes in the 9 cell-type populations (Supplementary Table 2). The expression of cell type marker genes is shown in the dot plot (Figure 1C). We observed 9 cell clusters (CD4T/NK cells: clusters 2, 3, 6, 14, 16, 28, 30, 32 and 37, expressing CD3D [19], CD7 [20], and IL7R (interleukin 7 receptor) [21]; CTLs: clusters 0, 4 and 20, expressing CD8A [22]; Endothelial cells: clusters 5, 15, 21, 22, 33, 39, 43 and 44, expressing vWF and CLDN5 [23, 24]; Fibroblasts/vSMCs: clusters 7, 8, 13, 17, 18, 19, 23, 29, 31 and 35, expressing LUM (Lumican) [25] and ACTA2 (alpha actin 2, smooth muscle); vSMCs: clusters 2, 5, 14, 15, 21, 28, 30, 34, 41 and 42, expressing ACTA2 (alpha actin 2, smooth muscle) [26, 27]; B/Plasma_B cells: clusters 9, 24 and 40, expressing CD79A [28]; Macrophages: clusters 1, 10, 11, 12, 25, 36 and 38, expressing FCGR3A [29]; Mast cells: cluster 27, expressing CPA3 [30]; Monocytes: clusters 34, expressing FCGR3B (https://panglaodb.se/); and DCs: clusters 26, 41 and 42, expressing CD1C [31], CLEC9A [32], LILRA4 (https://panglaodb.se/).

**Figure 2:**
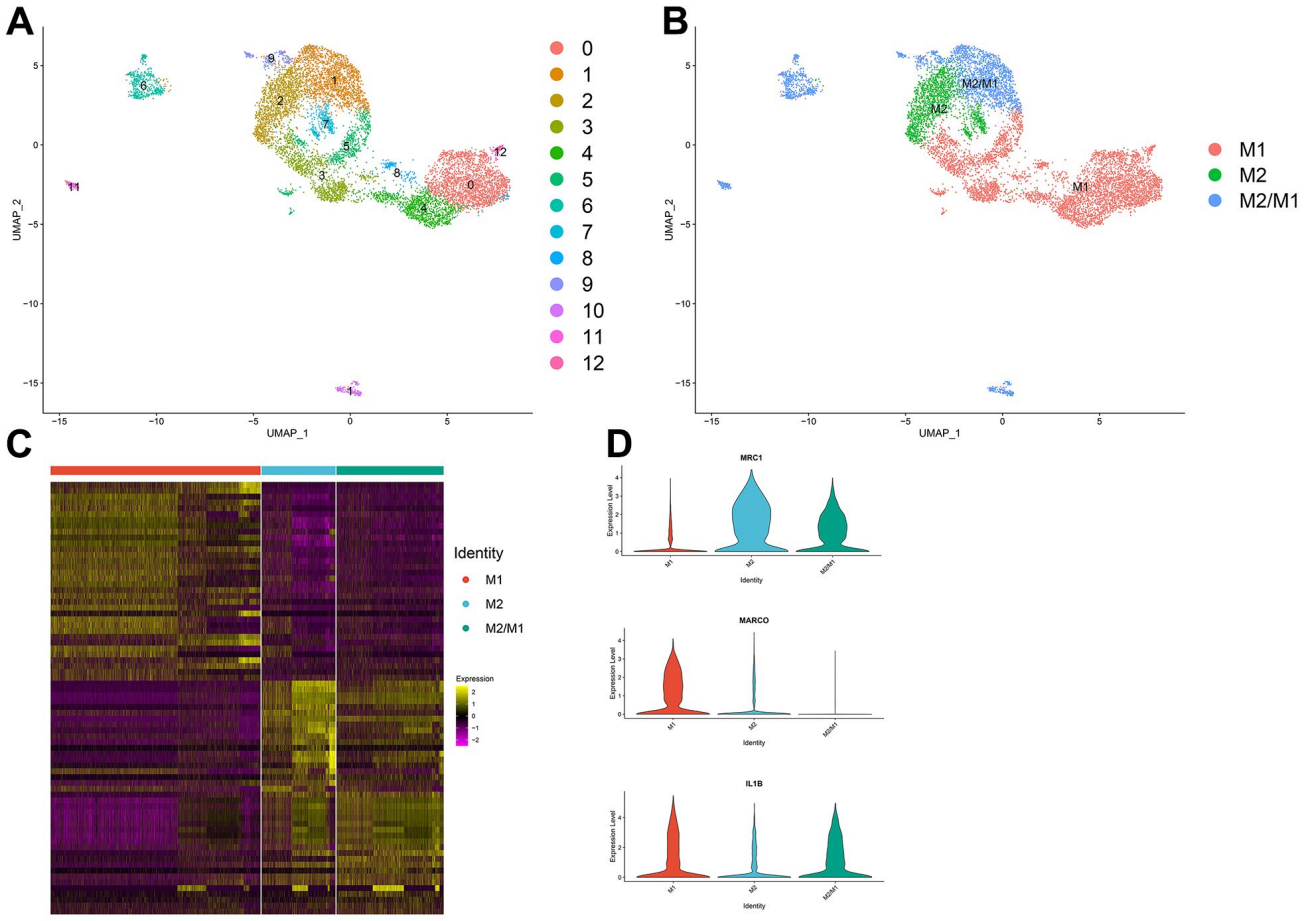
(**A**, **B**) The macrophage population was divided into 13 clusters, which were finally categorized into 3 cell populations (M1, M2, M2/M1). (**C**) Differential gene analysis among different subtypes of macrophages: The X-axis represents the macrophage subtypes, and the Y-axis represents the differentially expressed genes. (**D**) Violin plot of macrophage marker genes.

Thirteen clusters could be assigned to known cell lineages on the basis of marker gene expression (Figure 2A). We used t-SNE analysis to visualize the 9 clusters (Figure 2B) and identify marker genes in the 3 cell-type populations (Supplementary Table 3). We observed 3 cell clusters (M1: clusters 0, 3, 4, 5, 8 and 12, expressing MACRO [33] and IL1B [34]; M2: clusters 2, 7, expressing MRC1 [35, 36]; and M2/M1: clusters 1, 6, 9, 10 and 11, expressing both MRC1 and IL1B. Difference expressed genes analysis of the macrophage subtypes indicated that M2/M1 macrophages may be more similar to M2 macrophages than to M1 macrophages (Figure 2C, 2D).

### Macrophage trajectory analysis

**Figure 3:**
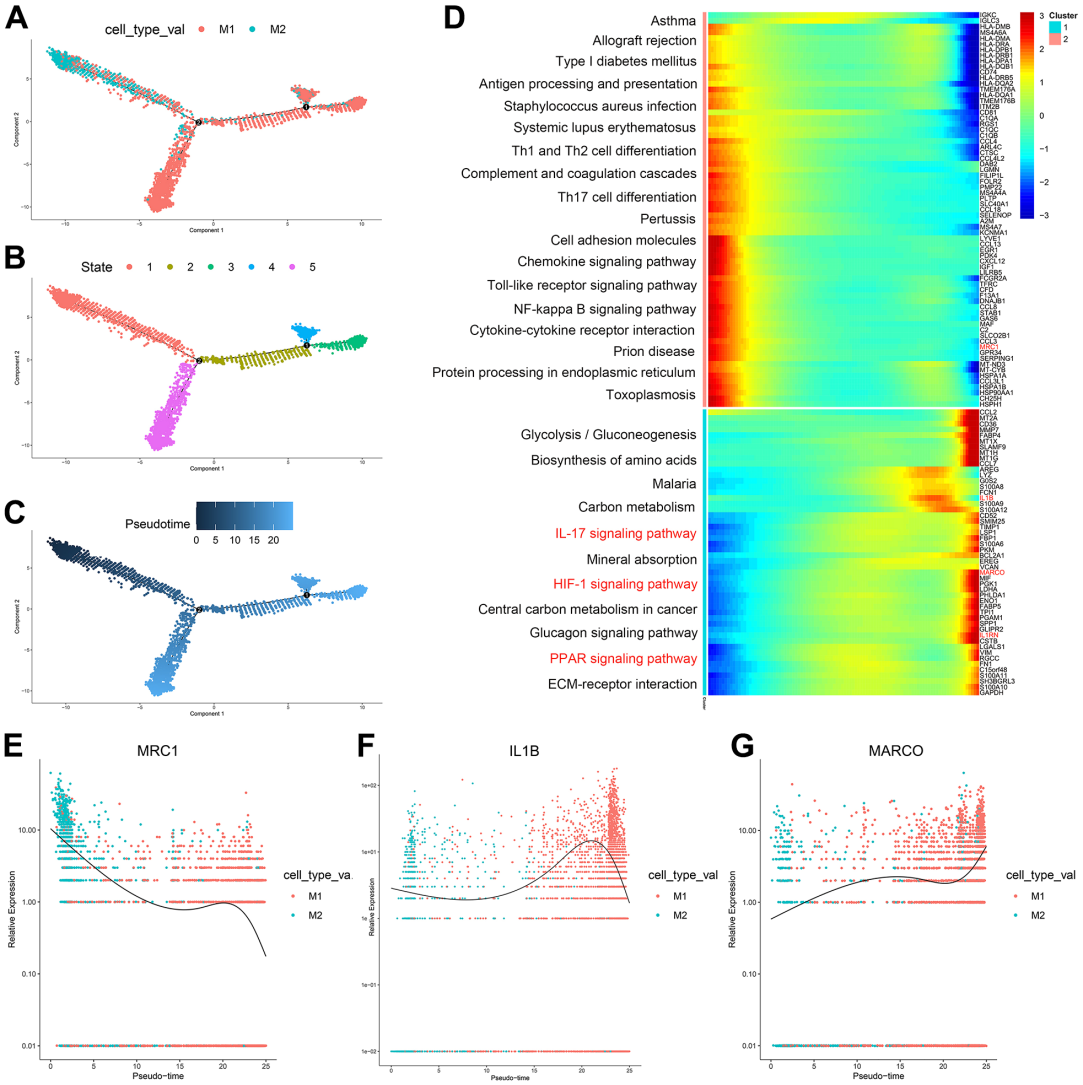
(**A**–**C**) Trajectory analysis of M1 macrophages and M2 macrophages. (**D**) The trajectory analysis of the heatmap of M1 macrophages versus M2 macrophages: The X-axis represents the timeline of trajectory analysis, the left Y-axis represents the KEGG enrichment results, and the right Y-axis represents the differentially expressed genes between the two clusters. (**E**–**G**) Pseudotime analysis of genes (MRC1, IL1B, and MARCO): The X-axis represents the cell of trajectory analysis, and the Y-axis represents the relative expression of the gene.

According to the trajectory analysis of M1 macrophages and M2 macrophages, we found that M2 macrophages can transform into M1 macrophages over time and that this trajectory can be divided into 5 states (Figure 3A–3C). By analyzing the cell trajectory (cell trajectory direction: from left to right), we divided the genes into two clusters. The expression of MRC1, the marker gene of M2 macrophages, decreased with time in cluster 2; MARCO and IL1B, the marker genes of M1 macrophages, increased with time in cluster 1 (Figure 3D–3G). KEGG analysis was performed on the genes of cluster 1, and a total of 11 signaling pathways were obtained. Among them, the IL17 signaling pathway, HIF-1 signaling pathway, and PPAR signaling pathway were closely related to the formation of atherosclerosis.

**Figure 4:**
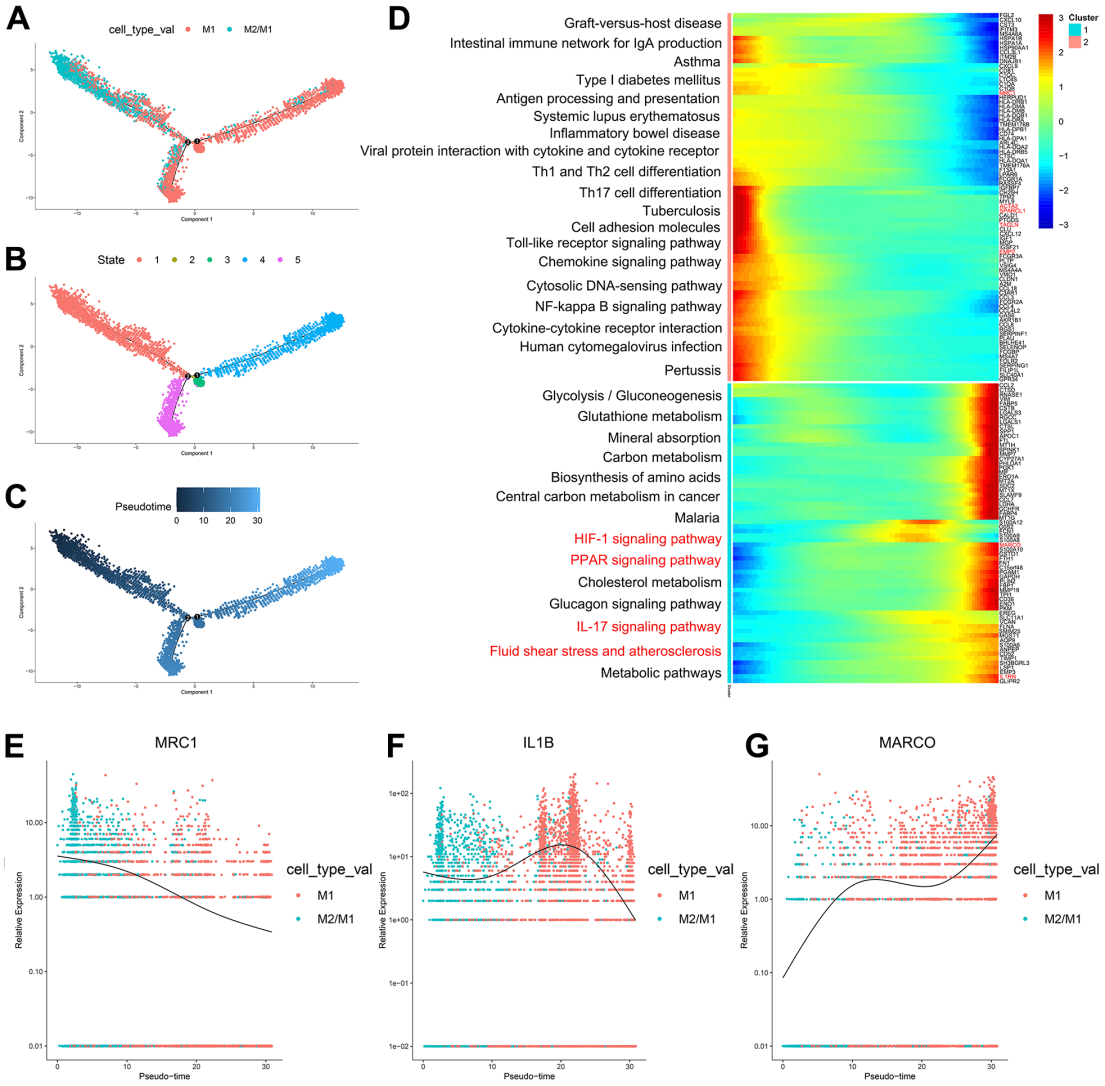
(**A**–**C**) Trajectory analysis of M1 macrophages and M2/M1 macrophages. (**D**) The trajectory analysis of the heatmap of M1 macrophages versus M2/M1 macrophages. The X-axis represents the timeline of trajectory analysis, the left Y-axis represents the KEGG enrichment results, and the right Y-axis represents the differentially expressed genes between the two clusters. (**E**–**G**) Pseudotime analysis of genes (MRC1, IL1B, and MARCO). The X-axis represents the cell of trajectory analysis, and the Y-axis represents the relative expression of the gene.

According to the trajectory analysis of M1 macrophages and M2/M1 macrophages, we found that M2/M1 macrophages can transform into M1 macrophages over time and that the trajectory could be divided into 5 states (Figure 4A–4C). By analyzing the cell trajectory (cell trajectory direction: from left to right), we divided the genes into two clusters. The expression of MRC1, the marker gene of M2 macrophages, decreased with time in cluster 2; MARCO and IL1B, the marker genes of M1 macrophages, increased with time in cluster 1 (Figure 4D–4G). KEGG analysis was performed on the genes of cluster 1, and a total of 11 signaling pathways were obtained. Among them, the IL17 signaling pathway, HIF-1 signaling pathway, PPAR signaling pathway and fluid shear stress and atherosclerosis were closely related to the formation of atherosclerosis.

**Figure 5:**
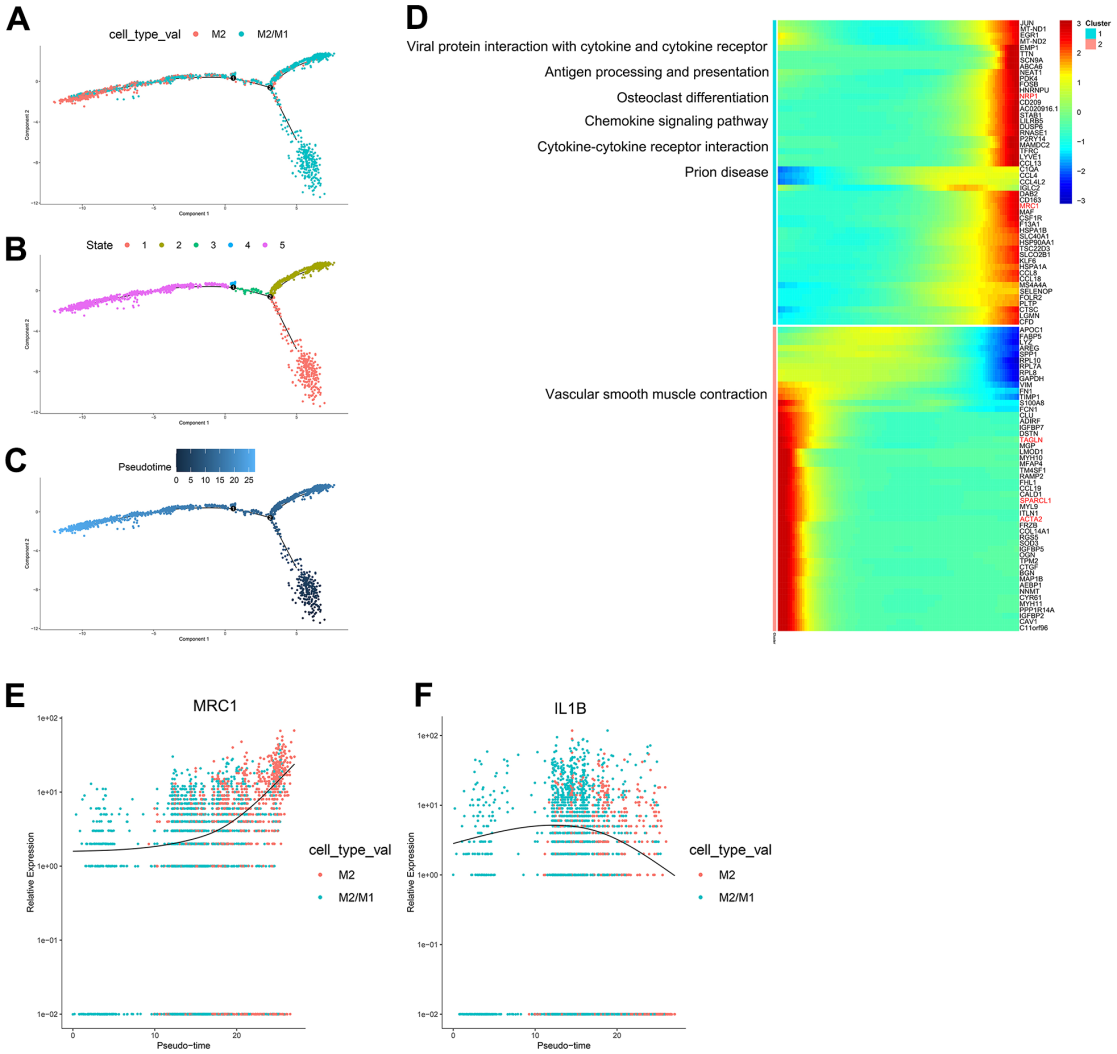
(**A**–**C**) Trajectory analysis of M2 macrophages and M2/M1 macrophages. (**D**) The trajectory analysis of the heatmap of M2 macrophages versus M2/M1 macrophages. The X-axis represents the timeline of trajectory analysis, the left Y-axis represents the KEGG enrichment results, and the right Y-axis represents the differentially expressed genes between the two clusters. (**E**, **F**) Pseudotime analysis of genes (MRC1, IL1B, and MARCO). The X-axis represents the cell of trajectory analysis, and the Y-axis represents the relative expression of the gene.

According to the trajectory analysis of M2 macrophages and M2/M1 macrophages, we found that M2/M1 macrophages can transform into M2 macrophages over time and that the trajectory could be divided into 5 states (Figure 5A–5C). By analyzing the cell trajectory (cell trajectory direction: from left to right), we divided the genes into two clusters. The expression of MRC1, the marker gene of M2 macrophages, decreased with time in cluster 2; IL1B, the marker gene of M1 macrophages, increased with time in cluster 1 (Figure 5D–5F). KEGG analysis was performed on the genes of cluster 1, and a total of 6 signaling pathways were obtained. These signaling pathways are mainly related to chemokines. In the single-cell transcriptome, IL1RN was upregulated during the transformation of M2 macrophages into M1 macrophages; SPARCL1, TAGLN and EMP2 were downregulated during the transformation of M2/M1 macrophages into M1 macrophages; IL-1RN was upregulated during the transformation from M2/M1 macrophages to M1 macrophages; NRP1 was upregulated during the transformation from M2/M1 macrophages to M2 macrophages; and SPARCL1, TAGLN and ACTA2 were downregulated during the transformation of M2/M1 macrophages into M2 macrophages.

### Disease prediction model results

**Figure 6:**
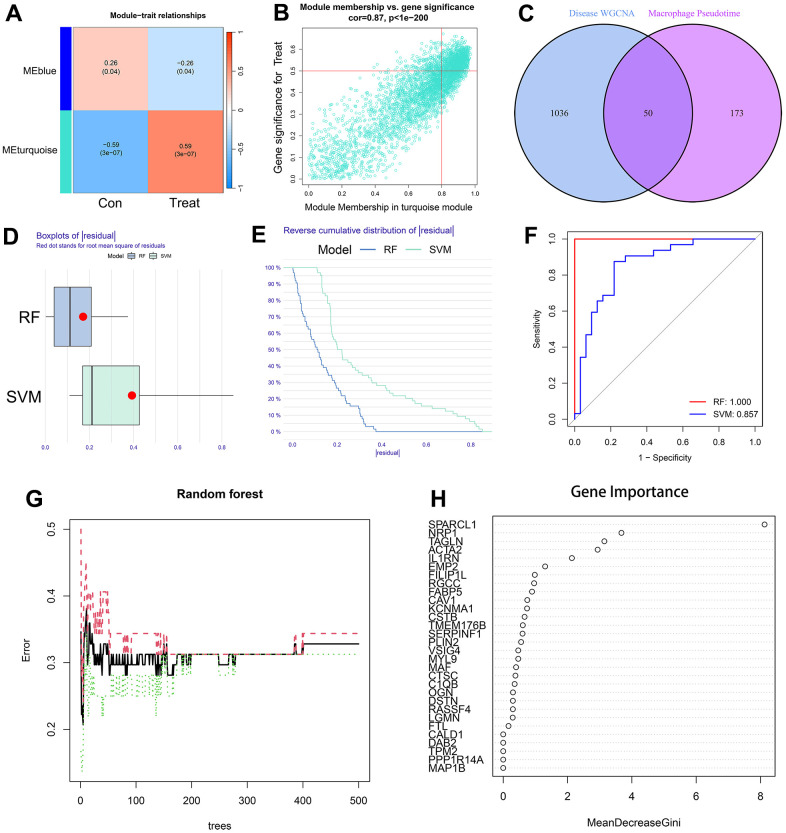
(**A**) The leftmost color block represents the module, and the rightmost color bar represents the correlation range. In the heatmap in the middle part, the darker the color is, the higher the correlation. Red indicates a positive correlation, and blue indicates a negative correlation. The numbers in each cell indicate relevance and significance. The X-axis represents the sample type. (**B**) A scatterplot of gene significance (GS) for treat vs. module membership in the turquoise module. There is a highly significant correlation between GS and MM in the module. (**C**) The left circle represents the disease-characteristic genes screened using the WGCNA method, and the right circle represents the characteristic genes that change most clearly over time between macrophage subtypes. The intersection of the two circles represents the intersecting genes. (**D**, **E**) Boxplot of the residual and reserve cumulative distribution of the residual. (**F**) The ROC curve shows the difference between SVM and RF. (**G**, **H**) RF analysis results and screening for important genes.

A total of 1086 AS-characteristic genes (module membership vs. gene significance cor=0.87, p<1e-200) from WGCNA and 223 DEGs in the macrophage trajectory analysis were obtained. Of these, we identified 50 intersecting genes (Figure 6A–6C). The comparison of the accuracy of SVM and RF in screening disease-characteristic genes indicated that the accuracy of RF was higher (Figure 6D–6F). We used the RF analysis method to obtain a total of 6 disease-characteristic genes (IL1RN, TAGLN, SPARCL1, NRP1, EMP2, and ACTA2) (Figure 6G, 6H).

**Figure 7:**
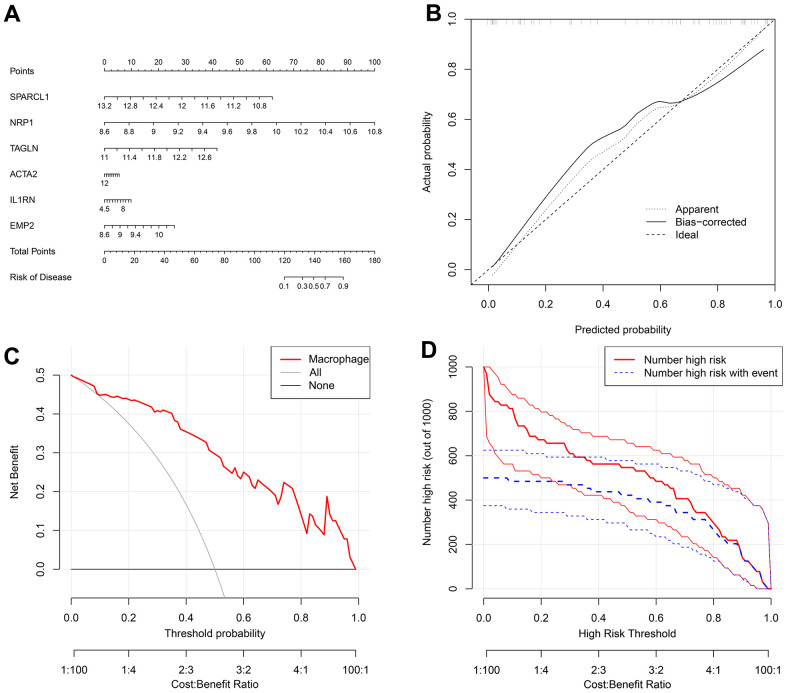
(**A**) A nomogram was created to represent the disease model. It uses the X-axis to display the expression of a single gene, as well as the score scale of a single gene, the total score scale of all genes, and the disease incidence scale. Meanwhile, the Y-axis shows individual genes, points, total points, and risk of disease. (**B**) A graph was used to plot the predicted event rate (Predicted Probability) on the abscissa and the observed actual event rate (Actual Rate) on the ordinate, ranging from 0 to 1. This can be interpreted as the event rate in percentage. The diagonal dashed line serves as the reference line, representing the scenario where the predicted value equals the actual value. (**C**) The DCA graph employs the threshold probability (ThresholdProbability) on the abscissa and the net profit rate after subtracting the disadvantages on the vertical axis. (**D**) A graph was used to represent the high-risk threshold and benefit rate on the abscissa, and the number of high risks on the ordinate.

The nomogram indicated that the probability of atherosclerosis increased to 90% when the total score of 6 disease-characteristic genes reached 160 and 10% when it reached 120 (Figure 7A). The calibration curve suggested that the model had reasonable accuracy in predicting the incidence of atherosclerosis (Figure 7B). DCA once again proved that the model has clinical utility (Figure 7C). The clinical impact curve showed that the benefit rate of the model was higher when the number of high-risk factors for atherosclerosis was smaller (Figure 7D).

**Figure 8:**
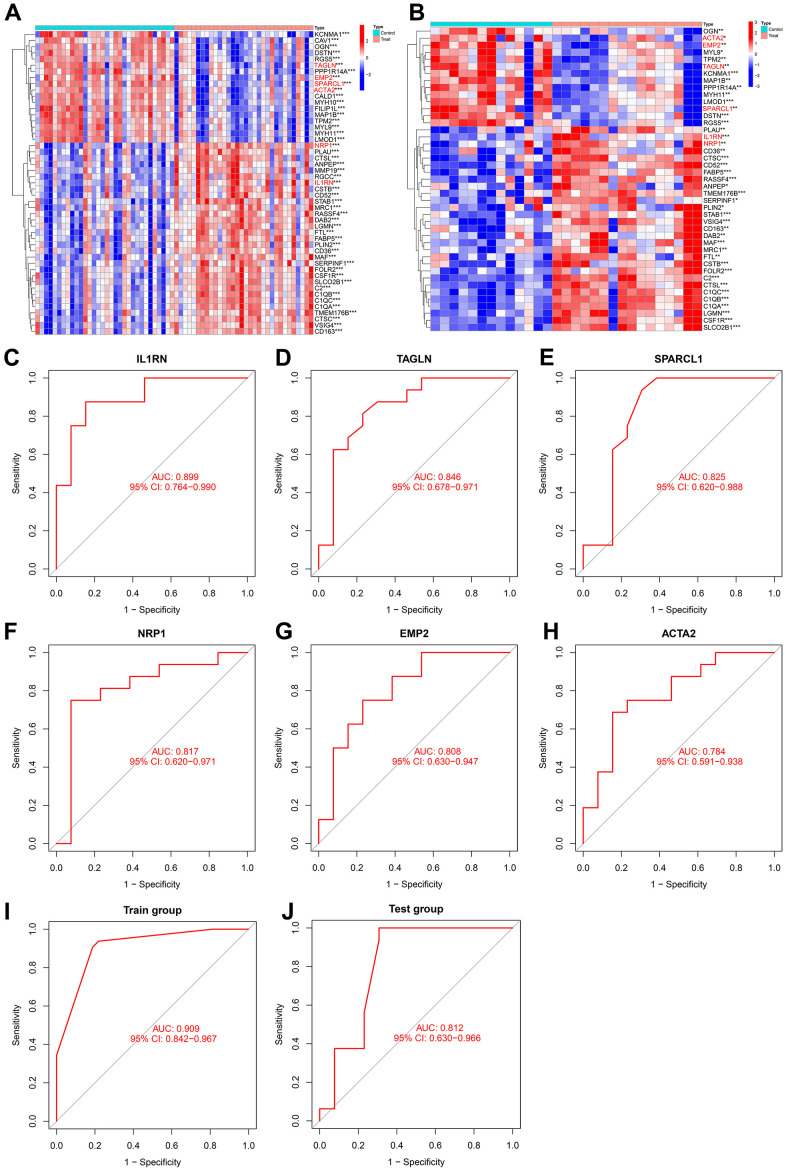
(**A**) Heatmap of intersecting genes in the training group. (**B**) Heatmap of intersecting genes in the test group. (**C**–**H**) ROC curves of disease signature genes in the test group. (**I**, **J**) The area under the curve of the AS prediction model in the train group and test group.

The NRP1 and IL1RN genes were significantly upregulated in both the training group and test group, while TAGLN, SPARCL1, EMP2 and ACTA2 were significantly downregulated in both the training group and test group (Figure 8A, 8B). The ROC curve values of the six genes in the test group were statistically significant (AUC (IL1RN): 0.899, 95% CI: 0.764-0.990; AUC (NRP1): 0.817, 95% CI: 0.620-0.971; AUC (TAGLN): 0.846, 95% CI: 0.678-0.971; AUC (SPARCL1): 0.825, 95% CI: 0.620-0.988; AUC (EMP2): 0.808, 95% CI: 0.630-0.947; AUC (ACTA2): 0.784, 95% CI: 0.591-0.938) (Figure 8C–8H). We constructed an AS prediction model using these six genes, which showed significant statistical significance in both the train group (AUC: 0.909, 95% CI: 0.842-0.967) and test group (AUC: 0.812, 95% CI: 0.630-0.966).

## DISCUSSION

Atherosclerosis plays an important role in cardiovascular disease. Its pathological mechanisms include dysfunction of the endothelial cell barrier, lipid accumulation, abnormal angiogenesis, and local chronic inflammatory reaction caused by immune cell infiltration (including T cells, B cells, myeloids), among others [37–39]. Among numerous immune cells, macrophages are undoubtedly the most important in the evolution of atherosclerosis, and their polarization acts as a double-edged sword in the occurrence and development of atherosclerosis [40]. The polarization of macrophages can also regulate the stability of plaques [41]. In our study, according to the results of pseudotime analysis, we found that M2 macrophages can transform into M1 macrophages, and M2/M1 (MRC1(+) IL1B (+)) macrophages can transform into M1 or M2 macrophages. According to the RF method, a total of six disease-characteristic genes of atherosclerosis were identified: in the single-cell transcriptome, IL1RN was upregulated during the transformation of M2 macrophages into M1 macrophages; SPARCL1, TAGLN and EMP2 were downregulated during the transformation of M2/M1 macrophages into M1 macrophages; IL-1RN was upregulated during the transformation from M2/M1 macrophages to M1 macrophages; NRP1 was upregulated during the transformation from M2/M1 macrophages to M2 macrophages; and SPARCL1, TAGLN and ACTA2 were downregulated during the transformation of M2/M1 macrophages into M2 macrophages. The changes in these genes in the bulk transcriptome were consistent.

The IL1RN gene has been found to have variable numbers of an 86 base pair (bp) tandem repeat in intron 2 [42], and this polymorphism is associated with various inflammatory diseases (systemic lupus erythematosus [43], type 2 diabetes mellitus (T2DM) [44]) and ischemic stroke [45]. A recent study showed that in thoracic aortic dissection, IL1RN^High^ macrophages, as a subtype of proinflammatory macrophages, are the main source of MMPs and inflammatory cytokines, which strengthens the formation of thoracic aortic aneurysm [46]. It was also predicted that these macrophages would be considered target cells for the treatment of patients with thoracic aortic aneurysm in the future. This is in line with our research results; in our study, M2 and M2/M1 macrophages were transformed into IL1RN^High^ M1 macrophages.

Neuron-1 (NRP1) belongs to the neurotransmitter family [47]. NRP1 is known to be overexpressed by macrophages in acute and chronic inflammation-related diseases (sepsis [48], type II diabetes [49, 50], and metabolic syndrome [51], etc.). Its function is mainly to act as a common receptor of Semaphorin 3A (Sema3A) and vascular endothelial growth factor a (VEGF-A165), thus regulating development and pathological angiogenesis, arteriogenesis and vascular permeability [52]. In atherosclerotic plaques, microvascular growth, or angiogenesis, destroys the stability of the plaque, thus increasing the risk of rupture [53]. In addition, some studies have proven (mainly in the context of cancer) that M2 macrophages can promote the formation of pathological microvessels [54, 55]. In our study, NRP1 was identified as a disease-characteristic gene of atherosclerosis, which indicates that NRP1^High^ M2 macrophages may be a major risk factor for the formation and stability of atherosclerosis.

According to previous studies, ACTA2 is a marker gene of vascular smooth muscle cells [56]. Some studies have shown that VSMCs can take up lipids and become foam cells [57], and undergo phenotypic transfer to macrophage like status in atherosclerotic plaques [58, 59]. In this study, we obtained ACTA2^High^ M2/M1 macrophages and found that they can transform into M1 and M2 macrophages and that the expression of ACTA2 decreased significantly during this period. This indicates that ACTA2^High^ M2/M1 macrophages may be involved in the development of atherosclerosis.

EMP2 is expressed at a high level in a large number of human tissues, including adult ovarian, heart, lung and intestinal tissues and fetal lung tissues [60]. The protein produced by EMP2 is a four-transmembrane protein that has been found to mediate a variety of vascular reactions [61]. In cancer-related studies, we observed that the higher the expression level of EMP2 in various tumor models *in vitro*, the more obvious the expression of pathological angiogenesis-related pathways was [62–65]. Some studies have also shown that the proportion of M1 macrophages in the placenta is significantly increased in EMP2 knockout rats [61]. Based on the above results, we boldly speculate that EMP2^High^ M2/M1 macrophages can not only promote the formation of pathological blood vessels, resulting in the destruction of plaque stability, but also become proinflammatory macrophages (EMP2^Low^ M1 macrophages) through phenotypic transformation.

Secreted acidic cysteine-rich protein-1 (SPARCL1) belongs to the matrix cell protein SPARC family and is an extracellular matrix (ECM) glycoprotein [66]. At present, research on SPARCL1 has mainly been performed in the context of cancers, including lung cancer [67, 68], prostate cancer [69], colon cancer [70], etc. Little is known about its function in the context of cardiovascular disease. One study showed that SPARCL1 can inhibit angiogenesis and support vascular morphogenesis and integrity [71]. We predict that SPACL1^High^ M2/M1 macrophages may play a role in inhibiting the formation of carotid atherosclerosis and maintaining the stability of carotid plaques by inhibiting angiogenesis.

It is well known that the smooth muscle cell marker TAGLN (SM22α) is downregulated in the pathogenesis of atherosclerosis, restenosis, abdominal aortic aneurysm and other arterial diseases [72]. Here, we speculate that TAGLN^High^ macrophages (in the M2/M1 macrophage subgroup) may be similar to ACTA2^High^ macrophages and originate from vascular smooth muscle cells. TAGLN^High^ M2/M1 macrophages can transform into TAGLN^Low^ M1 and TAGLN^Low^ M2 macrophages, and the expression of TAGLN decreases significantly during this period.

We identified 6 kinds of macrophages that may be related to the occurrence and development of atherosclerosis: IL1RN^High^ M1, IL1RN^High^ M1, NRP1^High^ M2, ACTA2^High^ M2/M1, EMP2^High^ M1/M1, SPACL1^High^ M2/M1 and TAGLN^High^ M2/M1 macrophages. This study is the first to use these macrophage marker genes to construct a model for predicting atherosclerosis. The expression trend of these genes in bulk transcriptome data is consistent with the expression trend of macrophage pseudotime analysis, which again proves the accuracy of these genes as disease-characteristic genes in atherosclerosis.

## CONCLUSIONS

IL1RN^High^ M1 macrophages, NRP1^High^ M2 macrophages, ACTA2^High^ M2/M1 macrophages, EMP2^High^ M1/M1 macrophages, SPACL1^High^ M2/M1 macrophages and TAGLN^High^ M2/M1 macrophages play a key role in the occurrence and development of arterial atherosclerosis. These macrophages may become target cells for treating atherosclerosis in the future. These macrophage marker genes can also be used to build a model to predict atherosclerosis.

## Supplementary Material

Supplementary Table 1

Supplementary Table 2

Supplementary Table 3

Supplementary Table 4

Supplementary Table 5

Supplementary Table 6

## References

[1] Biros E, Moran CS, Rush CM, Gäbel G, Schreurs C, Lindeman JH, Walker PJ, Nataatmadja M, West M, Holdt LM, Hinterseher I, Pilarsky C, Golledge J. Differential gene expression in the proximal neck of human abdominal aortic aneurysm. Atherosclerosis. 2014; 233:211–8. 10.1016/j.atherosclerosis.2013.12.017 24529146

[2] Winkels H, Ehinger E, Vassallo M, Buscher K, Dinh HQ, Kobiyama K, Hamers AAJ, Cochain C, Vafadarnejad E, Saliba AE, Zernecke A, Pramod AB, Ghosh AK, et al. Atlas of the Immune Cell Repertoire in Mouse Atherosclerosis Defined by Single-Cell RNA-Sequencing and Mass Cytometry. Circ Res. 2018; 122:1675–88. 10.1161/CIRCRESAHA.117.312513 29545366 PMC5993603

[3] Liu YC, Zou XB, Chai YF, Yao YM. Macrophage polarization in inflammatory diseases. Int J Biol Sci. 2014; 10:520–9. 10.7150/ijbs.8879 24910531 PMC4046879

[4] Chen W, Schilperoort M, Cao Y, Shi J, Tabas I, Tao W. Macrophage-targeted nanomedicine for the diagnosis and treatment of atherosclerosis. Nat Rev Cardiol. 2022; 19:228–49. 10.1038/s41569-021-00629-x 34759324 PMC8580169

[5] Groh L, Keating ST, Joosten LAB, Netea MG, Riksen NP. Monocyte and macrophage immunometabolism in atherosclerosis. Semin Immunopathol. 2018; 40:203–14. 10.1007/s00281-017-0656-7 28971272 PMC5809534

[6] Murray PJ, Allen JE, Biswas SK, Fisher EA, Gilroy DW, Goerdt S, Gordon S, Hamilton JA, Ivashkiv LB, Lawrence T, Locati M, Mantovani A, Martinez FO, et al. Macrophage activation and polarization: nomenclature and experimental guidelines. Immunity. 2014; 41:14–20. 10.1016/j.immuni.2014.06.008 25035950 PMC4123412

[7] Ruytinx P, Proost P, Van Damme J, Struyf S. Chemokine-Induced Macrophage Polarization in Inflammatory Conditions. Front Immunol. 2018; 9:1930. 10.3389/fimmu.2018.01930 30245686 PMC6137099

[8] Yang K, Xiao Q, Niu M, Pan X, Zhu X. Exosomes in atherosclerosis: Convergence on macrophages. Int J Biol Sci. 2022; 18:3266–81. 10.7150/ijbs.71862 35637946 PMC9134907

[9] Domschke G, Gleissner CA. CXCL4-induced macrophages in human atherosclerosis. Cytokine. 2019; 122:154141. 10.1016/j.cyto.2017.08.021 28899579

[10] Murray PJ, Wynn TA. Protective and pathogenic functions of macrophage subsets. Nat Rev Immunol. 2011; 11:723–37. 10.1038/nri3073 21997792 PMC3422549

[11] Mallat Z, Gojova A, Marchiol-Fournigault C, Esposito B, Kamaté C, Merval R, Fradelizi D, Tedgui A. Inhibition of transforming growth factor-beta signaling accelerates atherosclerosis and induces an unstable plaque phenotype in mice. Circ Res. 2001; 89:930–4. 10.1161/hh2201.099415 11701621

[12] Gong M, Zhuo X, Ma A. STAT6 Upregulation Promotes M2 Macrophage Polarization to Suppress Atherosclerosis. Med Sci Monit Basic Res. 2017; 23:240–9. 10.12659/msmbr.904014 28615615 PMC5484610

[13] Bisgaard LS, Mogensen CK, Rosendahl A, Cucak H, Nielsen LB, Rasmussen SE, Pedersen TX. Bone marrow-derived and peritoneal macrophages have different inflammatory response to oxLDL and M1/M2 marker expression - implications for atherosclerosis research. Sci Rep. 2016; 6:35234. 10.1038/srep35234 27734926 PMC5062347

[14] Chen L, Hong W, Ren W, Xu T, Qian Z, He Z. Recent progress in targeted delivery vectors based on biomimetic nanoparticles. Signal Transduct Target Ther. 2021; 6:225. 10.1038/s41392-021-00631-2 34099630 PMC8182741

[15] Langfelder P, Horvath S. WGCNA: an R package for weighted correlation network analysis. BMC Bioinformatics. 2008; 9:559. 10.1186/1471-2105-9-559 19114008 PMC2631488

[16] Xu D, Wang Y, Liu X, Zhou K, Wu J, Chen J, Chen C, Chen L, Zheng J. Development and clinical validation of a novel 9-gene prognostic model based on multi-omics in pancreatic adenocarcinoma. Pharmacol Res. 2021; 164:105370. 10.1016/j.phrs.2020.105370 33316381

[17] Vickers AJ, van Calster B, Steyerberg EW. A simple, step-by-step guide to interpreting decision curve analysis. Diagn Progn Res. 2019; 3:18. 10.1186/s41512-019-0064-7 31592444 PMC6777022

[18] Wang D, Burns R, Khalil M, Mei A, Hashemi E, Malarkannan S. Methods to Analyze the Developmental Trajectory of Human Primary NK Cells Using Monocle and SCENIC Analyses. Methods Mol Biol. 2022; 2463:81–102. 10.1007/978-1-0716-2160-8_7 35344169

[19] Noutsias M, Rohde M, Göldner K, Block A, Blunert K, Hemaidan L, Hummel M, Blohm JH, Lassner D, Kühl U, Schultheiss HP, Volk HD, Kotsch K. Expression of functional T-cell markers and T-cell receptor Vbeta repertoire in endomyocardial biopsies from patients presenting with acute myocarditis and dilated cardiomyopathy. Eur J Heart Fail. 2011; 13:611–8. 10.1093/eurjhf/hfr014 21422001

[20] Kim SH, McQueen PG, Lichtman MK, Shevach EM, Parada LA, Misteli T. Spatial genome organization during T-cell differentiation. Cytogenet Genome Res. 2004; 105:292–301. 10.1159/000078201 15237218

[21] Gupta B, Iancu EM, Gannon PO, Wieckowski S, Baitsch L, Speiser DE, Rufer N. Simultaneous coexpression of memory-related and effector-related genes by individual human CD8 T cells depends on antigen specificity and differentiation. J Immunother. 2012; 35:488–501. 10.1097/CJI.0b013e31826183a7 22735807

[22] Mitchell KG, Diao L, Karpinets T, Negrao MV, Tran HT, Parra ER, Corsini EM, Reuben A, Federico L, Bernatchez C, Dejima H, Francisco-Cruz A, Wang J, et al. Neutrophil expansion defines an immunoinhibitory peripheral and intratumoral inflammatory milieu in resected non-small cell lung cancer: a descriptive analysis of a prospectively immunoprofiled cohort. J Immunother Cancer. 2020; 8:e000405. 10.1136/jitc-2019-000405 32350118 PMC7213906

[23] Pusztaszeri MP, Seelentag W, Bosman FT. Immunohistochemical expression of endothelial markers CD31, CD34, von Willebrand factor, and Fli-1 in normal human tissues. J Histochem Cytochem. 2006; 54:385–95. 10.1369/jhc.4A6514.2005 16234507

[24] Chiba H, Ichikawa-Tomikawa N, Imura T, Sugimoto K. The region-selective regulation of endothelial claudin-5 expression and signaling in brain health and disorders. J Cell Physiol. 2021; 236:7134–43. 10.1002/jcp.30357 33694168

[25] Baba H, Ishiwata T, Takashi E, Xu G, Asano G. Expression and localization of lumican in the ischemic and reperfused rat heart. Jpn Circ J. 2001; 65:445–50. 10.1253/jcj.65.445 11348051

[26] Owens GK. Regulation of differentiation of vascular smooth muscle cells. Physiol Rev. 1995; 75:487–517. 10.1152/physrev.1995.75.3.487 7624392

[27] Guo DC, Papke CL, Tran-Fadulu V, Regalado ES, Avidan N, Johnson RJ, Kim DH, Pannu H, Willing MC, Sparks E, Pyeritz RE, Singh MN, Dalman RL, et al. Mutations in smooth muscle alpha-actin (ACTA2) cause coronary artery disease, stroke, and Moyamoya disease, along with thoracic aortic disease. Am J Hum Genet. 2009; 84:617–27. 10.1016/j.ajhg.2009.04.007 19409525 PMC2680995

[28] Liu Y, Barta SK. Diffuse large B-cell lymphoma: 2019 update on diagnosis, risk stratification, and treatment. Am J Hematol. 2019; 94:604–16. 10.1002/ajh.25460 30859597

[29] Ochoa MC, Minute L, Rodriguez I, Garasa S, Perez-Ruiz E, Inogés S, Melero I, Berraondo P. Antibody-dependent cell cytotoxicity: immunotherapy strategies enhancing effector NK cells. Immunol Cell Biol. 2017; 95:347–55. 10.1038/icb.2017.6 28138156

[30] Finlin BS, Zhu B, Confides AL, Westgate PM, Harfmann BD, Dupont-Versteegden EE, Kern PA. Mast Cells Promote Seasonal White Adipose Beiging in Humans. Diabetes. 2017; 66:1237–46. 10.2337/db16-1057 28250021 PMC5399616

[31] Heger L, Hofer TP, Bigley V, de Vries IJM, Dalod M, Dudziak D, Ziegler-Heitbrock L. Subsets of CD1c^+^ DCs: Dendritic Cell Versus Monocyte Lineage. Front Immunol. 2020; 11:559166. 10.3389/fimmu.2020.559166 33101275 PMC7554627

[32] Chen YP, Yin JH, Li WF, Li HJ, Chen DP, Zhang CJ, Lv JW, Wang YQ, Li XM, Li JY, Zhang PP, Li YQ, He QM, et al. Single-cell transcriptomics reveals regulators underlying immune cell diversity and immune subtypes associated with prognosis in nasopharyngeal carcinoma. Cell Res. 2020; 30:1024–42. 10.1038/s41422-020-0374-x 32686767 PMC7784929

[33] Davies LC, Jenkins SJ, Allen JE, Taylor PR. Tissue-resident macrophages. Nat Immunol. 2013; 14:986–95. 10.1038/ni.2705 24048120 PMC4045180

[34] Murray PJ. Macrophage Polarization. Annu Rev Physiol. 2017; 79:541–66. 10.1146/annurev-physiol-022516-034339 27813830

[35] Taylor PR, Martinez-Pomares L, Stacey M, Lin HH, Brown GD, Gordon S. Macrophage receptors and immune recognition. Annu Rev Immunol. 2005; 23:901–44. 10.1146/annurev.immunol.23.021704.115816 15771589

[36] Hesketh M, Sahin KB, West ZE, Murray RZ. Macrophage Phenotypes Regulate Scar Formation and Chronic Wound Healing. Int J Mol Sci. 2017; 18:1545. 10.3390/ijms18071545 28714933 PMC5536033

[37] Sun JX, Zhang C, Cheng ZB, Tang MY, Liu YZ, Jiang JF, Xiao X, Huang L. Chemerin in atherosclerosis. Clin Chim Acta. 2021; 520:8–15. 10.1016/j.cca.2021.05.015 34022243

[38] Poznyak A, Grechko AV, Poggio P, Myasoedova VA, Alfieri V, Orekhov AN. The Diabetes Mellitus-Atherosclerosis Connection: The Role of Lipid and Glucose Metabolism and Chronic Inflammation. Int J Mol Sci. 2020; 21:1835. 10.3390/ijms21051835 32155866 PMC7084712

[39] Geovanini GR, Libby P. Atherosclerosis and inflammation: overview and updates. Clin Sci (Lond). 2018; 132:1243–52. 10.1042/CS20180306 29930142

[40] Zheng Y, Qi B, Gao W, Qi Z, Liu Y, Wang Y, Feng J, Cheng X, Luo Z, Li T. Macrophages-Related Genes Biomarkers in the Deterioration of Atherosclerosis. Front Cardiovasc Med. 2022; 9:890321. 10.3389/fcvm.2022.890321 35845072 PMC9282674

[41] Eshghjoo S, Kim DM, Jayaraman A, Sun Y, Alaniz RC. Macrophage Polarization in Atherosclerosis. Genes (Basel). 2022; 13:756. 10.3390/genes13050756 35627141 PMC9142092

[42] Worrall BB, Azhar S, Nyquist PA, Ackerman RH, Hamm TL, DeGraba TJ. Interleukin-1 receptor antagonist gene polymorphisms in carotid atherosclerosis. Stroke. 2003; 34:790–3. 10.1161/01.STR.0000057815.79289.EC 12624309

[43] Wang Y, Su W, Li Y, Yuan J, Yao M, Su X, Wang Y. Analyzing the pathogenesis of systemic lupus erythematosus complicated by atherosclerosis using transcriptome data. Front Immunol. 2022; 13:935545. 10.3389/fimmu.2022.935545 35935949 PMC9354579

[44] Margaryan S, Kriegova E, Fillerova R, Smotkova Kraiczova V, Manukyan G. Hypomethylation of IL1RN and NFKB1 genes is linked to the dysbalance in IL1β/IL-1Ra axis in female patients with type 2 diabetes mellitus. PLoS One. 2020; 15:e0233737. 10.1371/journal.pone.0233737 32470060 PMC7259508

[45] Lee BC, Lee H, Park HK, Yang JS, Chung JH. Susceptibility for ischemic stroke in four constitution medicine is associated with polymorphisms of FCGR2A and IL1RN genes. Neurol Res. 2010 (Suppl 1); 32:43–7. 10.1179/016164109X12537002793922 20034444

[46] Liu X, Chen W, Zhu G, Yang H, Li W, Luo M, Shu C, Zhou Z. Single-cell RNA sequencing identifies an Il1rn^+^/Trem1^+^ macrophage subpopulation as a cellular target for mitigating the progression of thoracic aortic aneurysm and dissection. Cell Discov. 2022; 8:11. 10.1038/s41421-021-00362-2 35132073 PMC8821555

[47] Raimondi C. Neuropilin-1 enforces extracellular matrix signalling via ABL1 to promote angiogenesis. Biochem Soc Trans. 2014; 42:1429–34. 10.1042/BST20140141 25233427

[48] Dai X, Okon I, Liu Z, Wu Y, Zhu H, Song P, Zou MH. A novel role for myeloid cell-specific neuropilin 1 in mitigating sepsis. FASEB J. 2017; 31:2881–92. 10.1096/fj.201601238R 28325756 PMC5471517

[49] Moin ASM, Al-Qaissi A, Sathyapalan T, Atkin SL, Butler AE. Soluble Neuropilin-1 Response to Hypoglycemia in Type 2 Diabetes: Increased Risk or Protection in SARS-CoV-2 Infection? Front Endocrinol (Lausanne). 2021; 12:665134. 10.3389/fendo.2021.665134 34248841 PMC8261232

[50] Hoseini-Aghdam M, Sheikh V, Eftekharian MM, Rezaeepoor M, Behzad M. Enhanced expression of TIGIT but not neuropilin-1 in patients with type 2 diabetes mellitus. Immunol Lett. 2020; 225:1–8. 10.1016/j.imlet.2020.06.003 32540486

[51] Wilson AM, Shao Z, Grenier V, Mawambo G, Daudelin JF, Dejda A, Pilon F, Popovic N, Boulet S, Parinot C, Oubaha M, Labrecque N, de Guire V, et al. Neuropilin-1 expression in adipose tissue macrophages protects against obesity and metabolic syndrome. Sci Immunol. 2018; 3:eaan4626. 10.1126/sciimmunol.aan4626 29549139

[52] Roth L, Prahst C, Ruckdeschel T, Savant S, Weström S, Fantin A, Riedel M, Héroult M, Ruhrberg C, Augustin HG. Neuropilin-1 mediates vascular permeability independently of vascular endothelial growth factor receptor-2 activation. Sci Signal. 2016; 9:ra42. 10.1126/scisignal.aad3812 27117252

[53] Sluimer JC, Daemen MJ. Novel concepts in atherogenesis: angiogenesis and hypoxia in atherosclerosis. J Pathol. 2009; 218:7–29. 10.1002/path.2518 19309025

[54] Han C, Yang Y, Sheng Y, Wang J, Li W, Zhou X, Guo L. The mechanism of lncRNA-CRNDE in regulating tumour-associated macrophage M2 polarization and promoting tumour angiogenesis. J Cell Mol Med. 2021; 25:4235–47. 10.1111/jcmm.16477 33742511 PMC8093957

[55] Yang Y, Guo Z, Chen W, Wang X, Cao M, Han X, Zhang K, Teng B, Cao J, Wu W, Cao P, Huang C, Qiu Z. M2 Macrophage-Derived Exosomes Promote Angiogenesis and Growth of Pancreatic Ductal Adenocarcinoma by Targeting E2F2. Mol Ther. 2021; 29:1226–38. 10.1016/j.ymthe.2020.11.024 33221435 PMC7934635

[56] Chakraborty R, Saddouk FZ, Carrao AC, Krause DS, Greif DM, Martin KA. Promoters to Study Vascular Smooth Muscle. Arterioscler Thromb Vasc Biol. 2019; 39:603–12. 10.1161/ATVBAHA.119.312449 30727757 PMC6527360

[57] Choi HY, Rahmani M, Wong BW, Allahverdian S, McManus BM, Pickering JG, Chan T, Francis GA. ATP-binding cassette transporter A1 expression and apolipoprotein A-I binding are impaired in intima-type arterial smooth muscle cells. Circulation. 2009; 119:3223–31. 10.1161/CIRCULATIONAHA.108.841130 19528336

[58] Allahverdian S, Chehroudi AC, McManus BM, Abraham T, Francis GA. Contribution of intimal smooth muscle cells to cholesterol accumulation and macrophage-like cells in human atherosclerosis. Circulation. 2014; 129:1551–9. 10.1161/CIRCULATIONAHA.113.005015 24481950

[59] Bao Z, Li L, Geng Y, Yan J, Dai Z, Shao C, Sun Z, Jing L, Pang Q, Zhang L, Wang X, Wang Z. Advanced Glycation End Products Induce Vascular Smooth Muscle Cell-Derived Foam Cell Formation and Transdifferentiate to a Macrophage-Like State. Mediators Inflamm. 2020; 2020:6850187. 10.1155/2020/6850187 32831637 PMC7428884

[60] Wang YW, Cheng HL, Ding YR, Chou LH, Chow NH. EMP1, EMP 2, and EMP3 as novel therapeutic targets in human cancer. Biochim Biophys Acta Rev Cancer. 2017; 1868:199–211. 10.1016/j.bbcan.2017.04.004 28408326

[61] Chu A, Kok SY, Tsui J, Lin MC, Aguirre B, Wadehra M. Epithelial membrane protein 2 (Emp2) modulates innate immune cell population recruitment at the maternal-fetal interface. J Reprod Immunol. 2021; 145:103309. 10.1016/j.jri.2021.103309 33774530 PMC8722772

[62] Fu M, Maresh EL, Helguera GF, Kiyohara M, Qin Y, Ashki N, Daniels-Wells TR, Aziz N, Gordon LK, Braun J, Elshimali Y, Soslow RA, Penichet ML, et al. Rationale and preclinical efficacy of a novel anti-EMP2 antibody for the treatment of invasive breast cancer. Mol Cancer Ther. 2014; 13:902–15. 10.1158/1535-7163.MCT-13-0199 24448822 PMC4034757

[63] Gordon LK, Kiyohara M, Fu M, Braun J, Dhawan P, Chan A, Goodglick L, Wadehra M. EMP2 regulates angiogenesis in endometrial cancer cells through induction of VEGF. Oncogene. 2013; 32:5369–76. 10.1038/onc.2012.622 23334331 PMC3898317

[64] Qin Y, Takahashi M, Sheets K, Soto H, Tsui J, Pelargos P, Antonios JP, Kasahara N, Yang I, Prins RM, Braun J, Gordon LK, Wadehra M. Epithelial membrane protein-2 (EMP2) promotes angiogenesis in glioblastoma multiforme. J Neurooncol. 2017; 134:29–40. 10.1007/s11060-017-2507-8 28597184 PMC5695892

[65] Sun M, Cherian N, Liu L, Chan AM, Aguirre B, Chu A, Strawbridge J, Kim ES, Lin MC, Tsui I, Gordon LK, Wadehra M. Epithelial membrane protein 2 (EMP2) regulates hypoxia-induced angiogenesis in the adult retinal pigment epithelial cell lines. Sci Rep. 2022; 12:19432. 10.1038/s41598-022-22696-x 36371458 PMC9653491

[66] Gagliardi F, Narayanan A, Mortini P. SPARCL1 a novel player in cancer biology. Crit Rev Oncol Hematol. 2017; 109:63–8. 10.1016/j.critrevonc.2016.11.013 28010899

[67] Bendik I, Schraml P, Ludwig CU. Characterization of MAST9/Hevin, a SPARC-like protein, that is down-regulated in non-small cell lung cancer. Cancer Res. 1998; 58:626–9. 9485012

[68] Deng H, Hang Q, Shen D, Zhang Y, Chen M. Low expression of CHRDL1 and SPARCL1 predicts poor prognosis of lung adenocarcinoma based on comprehensive analysis and immunohistochemical validation. Cancer Cell Int. 2021; 21:259. 10.1186/s12935-021-01933-9 33980221 PMC8117659

[69] Xiang Y, Qiu Q, Jiang M, Jin R, Lehmann BD, Strand DW, Jovanovic B, DeGraff DJ, Zheng Y, Yousif DA, Simmons CQ, Case TC, Yi J, et al. SPARCL1 suppresses metastasis in prostate cancer. Mol Oncol. 2013; 7:1019–30. 10.1016/j.molonc.2013.07.008 23916135 PMC3838491

[70] Hu H, Wu D, Liu X, Yu H, Xu J, Cai W, Huang Y, Bai R, Zhang J, Gu Y, Zheng S, Ge W. SPARCL1 exhibits different expressions in left- and right-sided colon cancer and is downregulated via DNA methylation. Epigenomics. 2021; 13:1269–82. 10.2217/epi-2021-0231 34435512

[71] Regensburger D, Tenkerian C, Pürzer V, Schmid B, Wohlfahrt T, Stolzer I, López-Posadas R, Günther C, Waldner MJ, Becker C, Sticht H, Petter K, Flierl C, et al. Matricellular Protein SPARCL1 Regulates Blood Vessel Integrity and Antagonizes Inflammatory Bowel Disease. Inflamm Bowel Dis. 2021; 27:1491–502. 10.1093/ibd/izaa346 33393634 PMC8376124

[72] Chen R, Zhang F, Song L, Shu Y, Lin Y, Dong L, Nie X, Zhang D, Chen P, Han M. Transcriptome profiling reveals that the SM22α-regulated molecular pathways contribute to vascular pathology. J Mol Cell Cardiol. 2014; 72:263–72. 10.1016/j.yjmcc.2014.04.003 24735829

