# Supplementary material for: Integrating the characteristic genes of macrophage pseudotime analysis in single-cell RNA-seq to construct a prediction model of atherosclerosis
Source: Aging (Albany NY). 2023 Jul 8;15(13):6361–79. doi: 10.18632/aging.204856 (PMC10373969; doi:10.18632/aging.204856)
Supplement: Supplementary Table 6 [file aging-15-204856-s006.pdf]

Supplementary Table 6. The intersection of 50 macrophage-related genes.

S100A11
ACTA2
EMP2
MAF
TAGLN
NRP1
MYL9
CAV1
FTL
CALD1
DSTN
KCNMA1
DAB2
CSTB
TMEM176B
FILIP1L
LGMN
TPM2
PPP1R14A
SPARCL1
MAP1B
PLAU
RASSF4
RGS5
OGN
CTSC
MYH11
FOLR2
MMP19
C1QB
RGCC
CTSL
VSIG4
C1QC
CSF1R
SLCO2B1
C1QA
MYH10
STAB1
LMOD1
MRC1
SERPINF1
C2
CD163
ANPEP
PLIN2
CD52
IL1RN
FABP5
CD36
